# Enhancing Cardiopulmonary Function Through Spasticity Reduction—Effects of Botulinum Toxin A After Stroke: A Prospective Before–After Study

**DOI:** 10.3390/medicina62061066

**Published:** 2026-05-31

**Authors:** Laura Marieta Alexa, Alexandru-Cosmin Palcău, Livia Florentina Păduraru, Daniel Alexa, Claudia Andreea Palcău, Maria Magdalena Leon, Mihai Roca, Adriana Mihaela Ilieșiu, Lucia Corina Dima-Cozma

**Affiliations:** 1 Doctoral School, University of Medicine and Pharmacy “Grigore T. Popa”, 700115 Iași, Romania; 2 Neuromotor Rehabilitation Clinic, Clinical Rehabilitation Hospital, 700661 Iasi, Romania; 3 Faculty of Medicine, “Carol Davila” University of Medicine and Pharmacy, 050474 Bucharest, Romania; 4 Department of General Surgery, C.F. 2 Clinical Hospital, 011464 Bucharest, Romania; 5 Department of Cardiology, Elias Emergency University Hospital, 011461 Bucharest, Romania; 6 Department of Neurology, Clinical Rehabilitation Hospital, 700661 Iasi, Romania; 7 Faculty of Medicine, University of Medicine and Pharmacy “Grigore T. Popa”, 700111 Iași, Romania; 8 Department of Cardiology, Clinical Rehabilitation Hospital, 700661 Iasi, Romania; 9 Respiratory Rehabilitation Department, Clinical Rehabilitation Hospital, 700661 Iasi, Romania; 10 Department of Cardiology, “Prof. Dr. Theodor Burghele” Clinical Hospital, 050659 Bucharest, Romania

**Keywords:** post-stroke spasticity, botulinum toxin A, exercise capacity, cardiopulmonary function, stroke rehabilitation

## Abstract

*Background and Objectives*: Post-stroke spasticity is a frequent complication that contributes to impaired mobility, reduced functional independence, and decreased exercise tolerance. While botulinum toxin A (BoNT-A) is widely used to improve muscle tone, its effects on cardiopulmonary exercise capacity remain insufficiently characterized. This study aimed to evaluate the impact of BoNT-A treatment on cardiopulmonary performance and functional outcomes in patients with post-stroke spasticity. *Materials and Methods*: A prospective before–after study was conducted including 50 patients with post-stroke spasticity. Cardiopulmonary exercise testing was performed before and after BoNT-A administration. The primary outcome was peak oxygen consumption (VO_2_ peak), while secondary outcomes included anaerobic threshold (AT), exercise duration, maximal workload, 10 m walk test time, Barthel Index, and modified Rankin Scale (mRS). Paired comparisons and multivariable linear regression analyses were performed to assess changes and associated factors. *Results*: VO_2_ peak increased significantly following treatment (12.96 ± 2.70 vs. 13.55 ± 2.85 mL/kg/min; mean change 0.59 mL/kg/min, 95% CI 0.36–0.82; *p* < 0.001). Similar improvements were observed for AT (10.47 ± 2.77 vs. 10.97 ± 2.97 mL/kg/min; *p* < 0.001), exercise duration (6.70 ± 1.48 vs. 7.11 ± 1.55 min; *p* < 0.001), and maximal workload (44.70 ± 10.97 vs. 48.06 ± 12.60 W; *p* < 0.001). Functional performance improved, as indicated by reduced 10 m walk time (18.33 ± 4.67 vs. 17.36 ± 4.82 s; *p* < 0.001) and increased Barthel Index (57.62 ± 19.18 vs. 61.92 ± 21.10; *p* < 0.001). A modest but significant reduction in disability was observed on the mRS (*p* = 0.003). Baseline values were the strongest predictors of post-treatment outcomes, while smoking status was associated with worse walking performance. *Conclusions*: BoNT-A treatment was associated with modest but consistent improvements in cardiopulmonary exercise capacity and functional performance in patients with post-stroke spasticity. These findings suggest that spasticity management may be associated with functional and exercise-related benefits extending beyond local neuromuscular effects, although causal relationships cannot be established based on the present study design. Further controlled studies are needed to confirm these findings and evaluate their long-term clinical significance.

## 1. Introduction

Stroke continues to be one of the foremost causes of long-term disability globally, with motor deficits and spasticity playing a central role in diminishing functional independence and quality of life in stroke survivors [1,2]. Post-stroke spasticity affects a considerable proportion of patients and is linked to mobility impairment, elevated energy expenditure during movement, and decreased exercise tolerance [3,4]. Beyond its neuromuscular manifestations, spasticity may also exert an indirect influence on cardiovascular performance by raising the metabolic cost of movement and restricting engagement in physical activity [5].

Botulinum toxin type A (BoNT-A) is extensively employed as a first-line treatment for focal spasticity, with well-established evidence supporting its benefits in lowering muscle tone and enhancing both passive and active limb function [6]. BoNT-A exerts its therapeutic effect by inhibiting the presynaptic release of acetylcholine at the neuromuscular junction through cleavage of synaptosomal-associated protein 25 (SNAP-25), thereby reducing excessive muscle contraction and spastic hyperactivity. Through this localized chemodenervation effect, BoNT-A contributes to decreased muscle tone, improved passive movement, and enhanced functional mobility in patients with post-stroke spasticity [7,8]. Nevertheless, while its local neuromuscular effects are thoroughly documented [9], its potential influence on cardiopulmonary exercise capacity and functional performance has yet to be adequately investigated. Since post-stroke patients frequently present with diminished aerobic capacity and heightened cardiovascular risk, determining whether spasticity-targeted interventions can elicit systemic physiological changes during exercise carries considerable clinical significance [10].

Prior research has indicated that alleviating spasticity may enhance gait efficiency and functional mobility, potentially resulting in improved exercise tolerance [11]. However, evidence regarding the association between spasticity management and cardiopulmonary performance remains sparse, and the degree to which gains in neuromuscular function are accompanied by measurable improvements in cardiovascular efficiency has yet to be fully established.

Therefore, the purpose of the present study was to assess the indirect effects of BoNT-A treatment on cardiopulmonary exercise capacity, functional performance, and disability outcomes in patients with post-stroke spasticity. In addition, we aimed to identify clinical and demographic factors potentially associated with post-treatment outcomes.

## 2. Methods

### 2.1. Study Design and Population

This was a prospective, single-center, before–after observational study conducted in patients with post-stroke spasticity undergoing botulinum toxin A (BoNT-A) treatment. A total of 50 consecutive patients were included.

Eligible participants were adults (≥18 years) with a clinical diagnosis of ischemic stroke and persistent spasticity affecting the upper and/or lower limbs, in the subacute or chronic phase (≥3 months post-stroke). All patients were able to perform cardiopulmonary exercise testing and were capable of walking independently for at least 100 m, with or without the use of an assistive device. In addition, participants were required to be clinically stable and free from significant pain that could limit exercise performance.

Exclusion criteria included medications that could significantly interfere with heart rate response during exercise testing, including beta-blockers, non-dihydropyridine calcium channel blockers (e.g., verapamil or diltiazem), ivabradine, digoxin, and class I or III antiarrhythmic agents.; the presence of neurological disorders other than stroke or orthopedic conditions causing motor impairment (e.g., fractures, degenerative joint disease, or clinically significant hip or knee instability); severe psychiatric disorders; uncontrolled hypertension, cardiac arrhythmias, or unstable cardiovascular conditions; recent pulmonary embolism, subacute systemic illness or infection; and any contraindication to exercise testing or BoNT-A administration.

### 2.2. Intervention

All patients received intramuscular injections of botulinum toxin A (Dysport, Ipsen Pharma, Bucharest, Romania) for the treatment of focal spasticity. The injection protocol, including muscle selection and dosage, was individualized based on clinical evaluation and functional impairment. The most frequently targeted muscles included the gastrocnemius, soleus, tibialis posterior, biceps brachii, flexor carpi radialis, and finger flexors, depending on the individual spasticity pattern. Total BoNT-A doses were individualized according to clinical severity and distribution of spasticity (50–300 UI/muscle group). Injections were performed by experienced rehabilitation physicians using anatomical landmarks.

Follow-up assessments were initiated no earlier than 14 days after treatment, corresponding to the expected onset of the therapeutic effect of BoNT-A. Patients underwent repeated clinical and functional evaluations during follow-up, with the median reassessment interval occurring approximately 25–45 days after injection, a timeframe selected to capture the early therapeutic phase of treatment. During the study period, patients continued their conventional rehabilitation programs as prescribed by their treating physicians. Rehabilitation interventions were not standardized within the study protocol and could therefore vary between participants.

### 2.3. Study Variables and Outcome Measures

Demographic and clinical variables were collected for all participants, including age, sex, body mass index (BMI), and time since stroke (expressed in months). Cardiovascular risk factors were recorded as binary variables and included hypertension, diabetes mellitus, coronary artery disease, dyslipidemia, and smoking status (categorized as never, current, or former smoker). Cardiovascular risk factors were recorded as binary variables (presence/absence) to ensure consistency in data collection and to avoid excessive subgroup fragmentation, thereby maintaining adequate statistical power given the sample size.

Stroke location was determined based on available imaging data and categorized as left hemisphere, right hemisphere, or other. The “other” category included bilateral lesions and posterior circulation strokes (e.g., brainstem or cerebellar involvement). This categorization was adopted to avoid excessive subgroup fragmentation and to ensure adequate statistical power given the sample size.

Cardiopulmonary exercise capacity was assessed using a standardized cardiopulmonary exercise test. The primary outcome was peak oxygen consumption (VO_2_ peak, mL/kg/min). Secondary cardiopulmonary parameters included anaerobic threshold (AT, mL/kg/min), total exercise duration (minutes), and maximal workload achieved (watts).

Functional performance was evaluated using a 10 m walk test, with results expressed as time (s). Functional independence and disability were assessed using the Barthel Index and the modified Rankin Scale (mRS), respectively, both recorded at baseline and at follow-up after botulinum toxin A treatment [12].

Cardiopulmonary exercise testing was performed using a cycle ergometer with an incremental workload protocol, starting with a resting phase followed by a warm-up period and progressive increases in workload until maximal effort or symptom limitation. Participants were instructed to maintain a constant pedaling cadence throughout the test. Standard criteria for test termination were applied, including volitional exhaustion or the occurrence of clinically significant symptoms. Peak oxygen consumption (VO_2_ peak) and anaerobic threshold (AT) were determined using standard metabolic and ventilatory parameters.

The 10 m walk test was conducted under standardized conditions, with participants instructed to walk at a comfortable pace. The time required to complete the distance was recorded, and the best performance from repeated trials was used for analysis.

Functional scales (Barthel Index and modified Rankin Scale) were assessed by trained clinicians using standardized evaluation procedures.

All outcome measures were recorded at two time points: prior to botulinum toxin A administration (baseline) and at follow-up, allowing for within-subject comparison of changes over time.

### 2.4. Statistical Analysis

The data were analyzed using IBM SPSS Statistics version 25 and presented using Microsoft Office Excel/Word 2013. Continuous variables were tested for normality using the Shapiro–Wilk test. Normally distributed quantitative variables are presented as mean ± standard deviation (SD), while non-normally distributed variables are presented as median with interquartile range (IQR). Categorical variables are reported as counts and percentages.

Comparisons between independent groups were performed using the Mann–Whitney U test for non-normally distributed variables. The Kruskal–Wallis test was used for comparisons between more than two independent groups. Associations between categorical variables were assessed using Fisher’s exact test, with Bonferroni correction applied for multiple comparisons where appropriate. Correlations between non-normally distributed continuous variables were assessed using Spearman’s rank correlation coefficient.

Paired comparisons between pre- and post-treatment measurements were performed using the paired t-test for normally distributed variables. For non-normally distributed paired ordinal data (modified Rankin Scale), the Wilcoxon signed-rank test was used.

A multivariable linear regression model (ANCOVA approach) was subsequently performed for each post-intervention continuous outcome (VO_2_ peak, anaerobic threshold, exercise duration, maximal workload, 10 m walk test time, and Barthel Index), adjusting for baseline (pre-intervention) values and relevant clinical and demographic covariates, including age, sex, body mass index, time since stroke, stroke location, hypertension, diabetes mellitus, coronary artery disease, dyslipidemia, and smoking status. Statistical significance was set at *p* < 0.05. Given the exploratory nature of the study and the relatively limited sample size, multivariable regression analyses were considered hypothesis-generating and interpreted cautiously.

### 2.5. Ethical Considerations

The study was conducted in accordance with the principles of the Declaration of Helsinki and was approved by University of Medicine and Pharmacy “Grigore T. Popa” Ethics Committee (321/8 June 2023) and Iasi Clinical Recovery Hospital Ethics Committee (01/10 February 2023). Written informed consent was obtained from all participants prior to inclusion. 

## 3. Results

### 3.1. Main Characteristics of the Population

Fifty subjects were included in this study. Sex of the subjects was equally distributed (50%). The patients’ age followed a normal distribution (*p* = 0.166) had a mean of 74.02 (± 10.44) years. The BMI (body mass index) had a normal distribution (*p* = 0.151) and reported a mean of 26.76 (±5.14) kg/m^2^. Most of them (82%) had high blood pressure (HBP). Almost a third of them (36%) had diabetes mellitus (DM). Coronary artery disease (CAD) history was present in almost a fourth of them (28%). Over half of them (58%) had dyslipidemia and over a half of them (52%) being smokers, 10 (20%) being active smokers and 16 (32%) former smokers. Regarding stroke location, most patients had right-sided strokes (64.0%), followed by left-sided strokes (32.0%), while a small proportion presented with other or bilateral involvement (4.0%). Stroke duration until treatment onset did not follow a normal distribution (*p* < 0.001) and the median time was 8.5 (4.0–13.25) months. The median follow-up assessments were performed at 25–45 days after treatment (Table 1).

### 3.2. Peak Oxygen Uptake (VO_2_ Peak)

**Figure 1:**
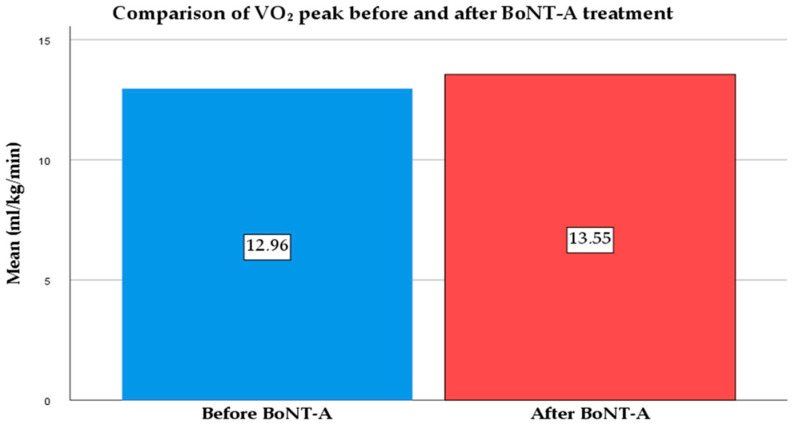
Comparison of VO_2_ peak before and after BoNT-A treatment.

The VO_2_ peak before the treatment had a normal distribution (*p* = 0.431) with an average of 12.96 (±2.70) ml/kg/min. The VO_2_ peak after the treatment had a normal distribution (*p* = 0.789) with an average of 13.55 (±2.85) ml/kg/min. VO_2_ peak increased significantly following treatment (12.96 ± 2.70 vs. 13.55 ± 2.85 mL/kg/min), with a mean improvement of 0.59 mL/kg/min (95% CI: 0.36–0.82, *p* < 0.001) (Figure 1).

A multivariable linear regression model was performed to evaluate factors associated with post-intervention values, adjusting for baseline (pre-intervention) measurements. The overall model was statistically significant (R^2^ = 0.934, adjusted R^2^ = 0.915, *p* < 0.001). Baseline VO_2_ peak was the only independent predictor of post-treatment values (B = 0.983, *p* < 0.001). No significant associations were found for age, sex, BMI, time since stroke, stroke location, or comorbidities (all *p* > 0.05).

### 3.3. Anaerobic Threshold (AT)

**Figure 2:**
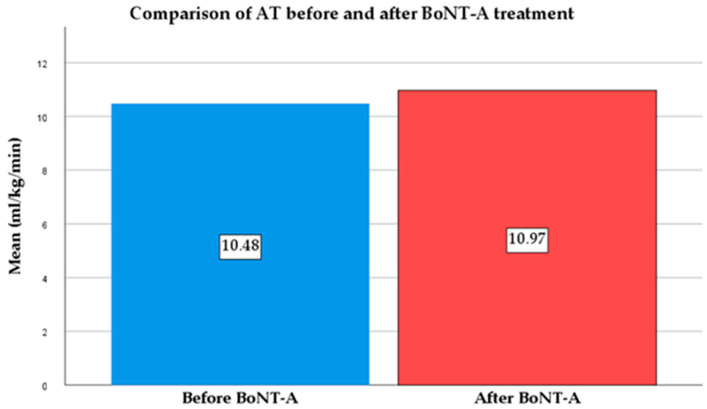
Comparison of AT before and after BoNT-A treatment.

The AT before the treatment had a normal distribution (*p* = 0.240) with an average of 10.47 (±2.77) ml/kg/min. The AT after the treatment had a normal distribution (*p* = 0.196) with an average of 10.97 (±2.97) ml/kg/min. AT increased significantly following treatment (10.47 ±2.77 vs. 10.97 ±2.97 mL/kg/min), with a mean improvement of 0.49 mL/kg/min (95% CI: 0.28–0.70, *p* < 0.001) (Figure 2).

A multivariable linear regression model (ANCOVA) was performed to evaluate factors associated with post-treatment anaerobic threshold. The overall model was statistically significant (R^2^ = 0.954, adjusted R^2^ =0.937, *p* < 0.001). Baseline anaerobic threshold was a strong independent predictor of post-treatment values (B = 1.003, *p* < 0.001). In addition, right-sided stroke was independently associated with higher post-treatment anaerobic threshold compared to left-sided stroke (B = 0.825, *p* = 0.010). No significant differences were observed for other stroke locations or for demographic and clinical variables (all *p* > 0.05).

### 3.4. Exercise Duration

**Figure 3:**
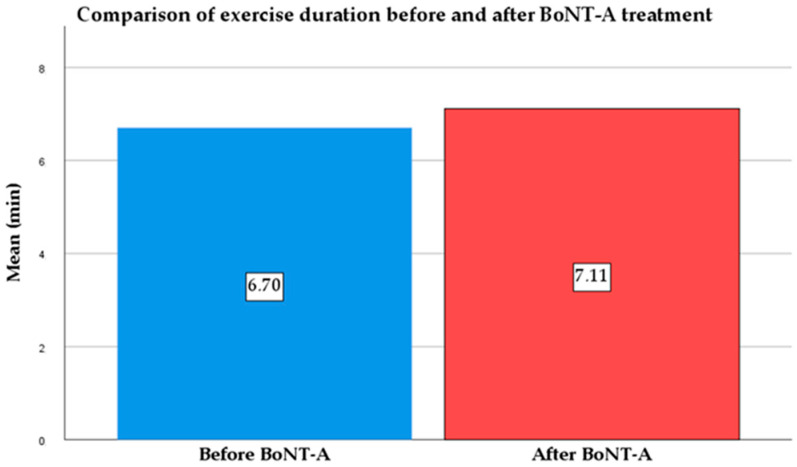
Comparison of exercise duration before and after BoNT-A treatment.

The exercise duration before the treatment had a normal distribution (*p* = 0.246) with an average of 6.70 (±1.48) min. The exercise duration after the treatment had a normal distribution (*p* = 0.849) with an average of 7.11 (±1.55) min. Exercise increased significantly following treatment (6.70 ±1.48 vs. 7.11 ±1.55 min), with a mean improvement of 0.41 min (95% CI: 0.24–0.58, *p* < 0.001) (Figure 3).

A multiple linear regression model was performed to identify predictors of post-intervention exercise duration. The model was statistically significant (F(13,36) = 22.46, *p* < 0.001) and explained 89% of the variance (R^2^ = 0.89; adjusted R^2^ = 0.85). Baseline exercise duration was the strongest predictor of post-intervention performance (B = 0.997, β = 0.95, *p* < 0.001). None of the demographic or clinical variables showed independent associations with the outcome.

### 3.5. Maximum Workload

**Figure 4:**
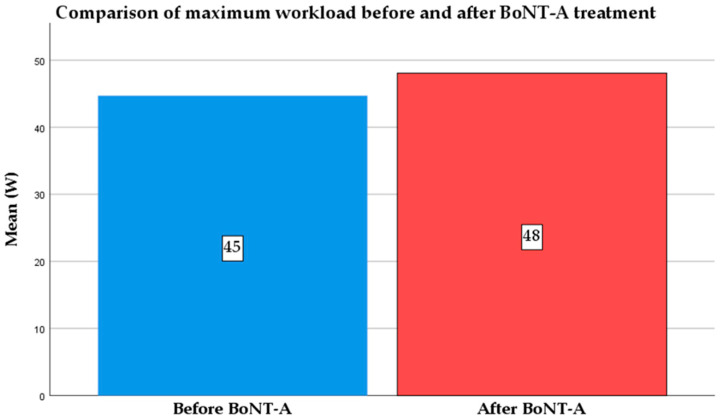
Comparison of maximum workload before and after BoNT-A treatment.

The maximum workload before the treatment had a normal distribution (*p* = 0.895) with an average of 44.70 (±10.97) W. The maximum workload after the treatment had a normal distribution (*p* = 0.265) with an average of 48.06 (±12.60) W. Maximum workload increased significantly following treatment (44.70 ±10.97 vs. 48.06 ±12.60 W), with a mean improvement of 3.36 W (95% CI: 2.01–4.70, *p* < 0.001) (Figure 4).

A multiple linear regression model was performed to identify predictors of post-intervention maximal workload. The model was statistically significant (F(13,36) = 20.28, *p* < 0.001) and explained 88% of the variance (R^2^ = 0.88; adjusted R^2^ = 0.84). Baseline maximal workload was the strongest predictor of post-intervention performance (B = 1.07, β = 0.93, *p* < 0.001). None of the demographic or clinical variables were independently associated with the outcome.

### 3.6. 10 m Walk Time

**Figure 5:**
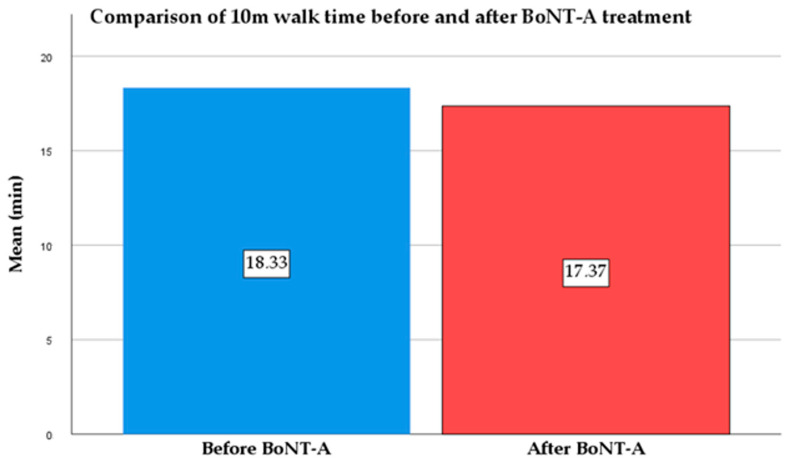
Comparison of 10 m walk time before and after BoNT-A treatment.

The 10 m walk time before the treatment had a normal distribution (*p* = 0.367) with an average of 18.33 (±4.67) sec. The 10 m walk time after the treatment had a normal distribution (*p* = 0.265) with an average of 17.36 (±4.82) sec. 10 m walk time decreased significantly following treatment (18.33 ± 4.67 vs. 17.36 ± 4.82 s), with a mean improvement of 0.97 s (95% CI: 0.67–1.27, *p* < 0.001) (Figure 5).

In the multiple linear regression model for 10 m walk test time post-intervention, the model was statistically significant (F(13,36) = 83.52, *p* < 0.001) and explained 96.8% of the variance in the dependent variable (R^2^ = 0.968; adjusted R^2^ = 0.956). The strongest predictor of post-intervention walking time was baseline 10 m walk test time (B = 0.995; β = 0.966; *p* < 0.001). Among the clinical and demographic variables, diabetes mellitus was associated with a small but statistically significant decrease in post-intervention 10 m walk time, indicating slightly faster performance after adjustment for other covariates (B = −0.926; *p* = 0.009). In contrast, both former and current smoking status were independently associated with increased walking time, suggesting worse functional mobility compared to non-smokers, with a delay of approximately 0.8–0.9 s in post-intervention performance (B = 0.830, *p* = 0.024; and B = 0.875, *p* = 0.046, respectively). All other variables included in the model did not show statistically significant associations with the outcome.

### 3.7. Barthel Index

**Figure 6:**
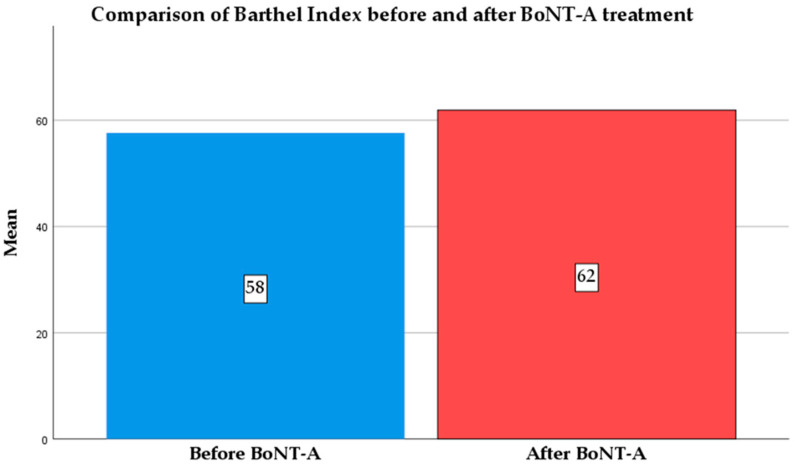
Comparison of Barthel Index before and after BoNT-A treatment.

The Barthel Index before the treatment had a normal distribution (*p* = 0.069) with an average of 57.62 (±19.18). The Barthel Index after the treatment had a normal distribution (*p* = 0.210) with an average of 61.92 (±21.10). The Barthel Index increased significantly following treatment (57.62 ± 19.18 vs. 61.92 ± 21.10), with a mean improvement of 4.3 (95% CI: 2.46–6.13, *p* < 0.001) (Figure 6).

In the multiple linear regression model predicting Barthel Index post-intervention, the model was statistically significant (F(13,36) = 35.21, *p* < 0.001) and explained 92.7% of the variance in functional outcome (R^2^ = 0.927; adjusted R^2^ = 0.901). The strongest predictor of post-intervention Barthel Index was baseline Barthel Index (B = 1.059; β = 0.963; *p* < 0.001), indicating that functional status after intervention was almost entirely determined by initial functional status. None of the clinical or demographic variables reached statistical significance, although sex (*p* = 0.085) and current smoking status (*p* = 0.086) showed borderline negative associations with post-intervention functional independence.

### 3.8. Modified Rankin Scale (mRS)

**Figure 7:**
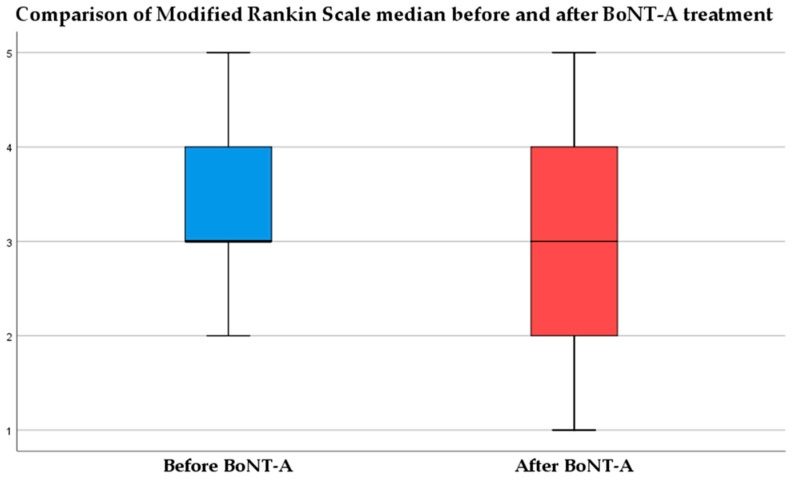
Comparison of mRS median before and after BoNT-A treatment.

The mRS before the treatment had a non-normal distribution (*p* < 0.001) with a median of 3 (3–5). The mRS after the treatment had a non-normal distribution (*p* < 0.001) with a median of 3 (2–4). The analysis showed a statistically significant improvement in functional outcome following treatment (Z = −2.985, *p* = 0.003), with 18 patients demonstrating lower mRS scores after treatment, 4 patients showing higher scores, and 28 patients remaining unchanged. In the multiple linear regression model predicting modified Rankin Scale (mRS) score after treatment, the model was statistically significant (F(13,36) = 11.84, *p* < 0.001) and explained 81.0% of the variance in post-treatment functional outcome (R^2^ = 0.810; adjusted R^2^ = 0.742) (Figure 7).

The strongest predictor of post-treatment mRS was baseline mRS score (B = 0.953; β = 0.807; *p* < 0.001), indicating that initial functional status strongly determined post-treatment disability level. None of the clinical or demographic variables were statistically significant predictors of post-treatment mRS in the adjusted model, although stroke duration showed a borderline association (*p* = 0.081).

## 4. Discussion

The findings of the present study contribute to a growing, yet still limited, body of evidence suggesting that focal spasticity management with BoNT-A may exert beneficial effects beyond the local, neuromuscular level. Across multiple outcome measures, we observed a modest, but consistent and statistically significant improvements following treatment, encompassing aerobic exercise capacity, walking performance, and functional independence. A smaller, though detectable, reduction in global disability was also recorded.

### 4.1. Cardiopulmonary Exercise Capacity

The improvements in cardiopulmonary exercise parameters observed in our cohort are of particular interest, given the scarcity of direct evidence in this domain. BoNT-A does not act on cardiovascular physiology directly. However, by reducing abnormal muscle tone, it may lower the mechanical and metabolic cost of movement, potentially permitting patients to sustain exercise at greater intensities or for longer durations [13]. Studies conducted in children with cerebral palsy have demonstrated that BoNT-A injection into spastic muscles not only reduced spasticity and improved gait pattern, but also reduced energy consumption, resulting in functional improvement [14]. This suggests that the link between spasticity reduction and metabolic efficiency of movement may not be only applied to the stroke population. In our cohort, the concurrent improvements in exercise duration and maximal workload further support the opinion that functional gains following spasticity treatment may extend beyond local effects and influence systemic exercise capacity. Importantly, the relatively short follow-up interval likely reflects early physiological and functional consequences of reduced spasticity rather than true long-term cardiopulmonary conditioning or cardiovascular adaptation. The observed improvements may therefore represent early indicators of enhanced movement efficiency and reduced metabolic demand during exercise.

### 4.2. Walking Performance and Functional Mobility

Our results regarding functional mobility are broadly consistent with prior randomized evidence. A systematic review examining BoNT-A for lower limb spasticity found that two studies reported significant improvements in gait velocity, and one study showed significant improvement in two-minute walking distance [15]. The overall evidence of the review was considered insufficiently robust to support widespread reimbursement at the time. More recently, a randomized controlled trial demonstrated that robotic gait training performed 2 weeks after botulinum toxin injection improved walking ability [16], with the greatest benefit observed in patients with intermediate baseline walking speed. This finding underscores both the importance of patient selection and the potential value of combining pharmacological and rehabilitative approaches. Our observation of improved 10 m walk test performance aligns with these results, reinforcing the argument that spasticity-targeted treatment can translate into clinically meaningful mobility gains.

### 4.3. Functional Independence

Improvements in Barthel Index scores further support the clinical relevance of BoNT-A therapy. These findings are consistent with evidence from a systematic review of early post-stroke spasticity interventions, in which the Modified Barthel Index was the most frequently employed activity-domain outcome measure, with several studies reporting significant improvements following spasticity-reducing interventions [17]. A large network meta-analysis demonstrated that multiple physical rehabilitation modalities targeting post-stroke upper limb spasticity were associated with meaningful gains in activities of daily living, as measured by the Modified Barthel Index. Improvements in functional independence are a consistent finding across diverse spasticity-focused treatment approaches, regardless of the specific modality employed [18]. Taken together, these data suggest that reductions in muscle tone achieved pharmacologically or through physical rehabilitation are reliably associated with measurable downstream gains in daily function, even if the magnitude of such changes remains moderate.

### 4.4. Global Disability Outcomes

The reduction in global disability as measured by the modified Rankin Scale was smaller and less pronounced compared to other outcomes. As noted in the literature, with a limited number of levels, the mRS may be less responsive to change than some other stroke scales [19,20]. Furthermore, the scale aims to measure overall disability and does not provide specific details on particular deficits or impairments [21,22], rendering it inherently less sensitive to domain-specific improvements such as those conferred by focal spasticity treatment. In contrast, more domain-specific outcome measures, such as walking performance tests or cardiopulmonary exercise parameters, may be more sensitive to subtle functional changes occurring shortly after focal spasticity treatment, which could explain the discrepancy between the magnitude of improvements observed across different outcome measures. The 2024 clinical practice guideline for stroke rehabilitation reflected a similar concern, noting a downgrade in the strength of recommendation for BoNT-A and acknowledging that its efficacy is dependent on patient characteristics and preferences [23].

### 4.5. Predictors of Post-Treatment Outcomes

Across all regression models, pre-treatment values consistently emerged as the strongest determinants of post-treatment outcomes. This is well established in the stroke rehabilitation literature. Systematic reviews have identified functional status on admission as one of the most impactful prognostic factors in inpatient stroke rehabilitation [24] and the dominance of baseline performance in our models is therefore expected. It does not, however, diminish the clinical relevance of the observed treatment effects. Beyond baseline status, few additional variables emerged as independent predictors. Two exceptions warrant discussion. First, smoking was associated with worse post-treatment walking performance, a finding consistent with evidence showing that lifestyle-related risk factors such as smoking may exacerbate stroke severity and further complicate recovery [25,26]. Second, diabetes showed a modest association with improved walking time after covariate adjustment. This finding is biologically counterintuitive and inconsistent with the broader literature, which generally associates diabetes mellitus with poorer functional recovery after stroke [27,28]. Therefore, this observation should be interpreted cautiously and considered hypothesis-generating rather than evidence of a true beneficial association. Residual confounding, regression to the mean, and model instability related to the relatively small sample size may have contributed to this result.

An additional finding of interest was the association between right-hemisphere stroke localization and higher post-treatment anaerobic threshold values. Although the mechanisms underlying this observation remain uncertain, hemispheric differences in motor control, autonomic regulation, spatial attention, and post-stroke functional recovery have previously been proposed as potential contributors to variability in rehabilitation outcomes after stroke [29,30]. Nevertheless, given the relatively small subgroup sizes and exploratory nature of the analysis, this finding should be interpreted cautiously and requires confirmation in larger studies.

### 4.6. Clinical Implications

Collectively, the present findings support the view that spasticity management represents a clinically meaningful component of post-stroke rehabilitation. Its effects appear to extend beyond local muscle tone reduction to encompass systemic exercise capacity, functional mobility, and independence in daily activities. These results have direct implications for rehabilitation planning. Emerging evidence on combined interventions suggests that integrating BoNT-A therapy with structured exercise programs and task-specific training may potentiate functional outcomes beyond what pharmacological treatment can achieve alone.

Stroke survivors frequently present with multiple cardiovascular comorbidities, including hypertension, coronary artery disease, diabetes mellitus, and dyslipidemia, all of which may negatively influence exercise tolerance, rehabilitation potential, and long-term prognosis [31].

Although the magnitude of improvement in VO_2_ peak and walking performance was modest, even small functional gains may be clinically relevant in selected post-stroke patients with limited baseline exercise capacity. Nevertheless, the long-term clinical significance of these changes remains uncertain and warrants further investigation.

The clinical significance of the observed improvements should also be interpreted in the context of reported minimal clinically important difference (MCID) thresholds. For the Barthel Index, published MCID estimates in stroke populations was 5 points in Anchor-Based Method and 4 points in Distribution Based Method (1.85–4.73), but it depends on patient characteristics and study methodology [32,33]. The mean improvement observed in our cohort (4.3 points) therefore approached the range of clinically meaningful change.

In contrast, although walking performance improved significantly, the estimated increase in gait speed remained below commonly proposed MCID thresholds for post-stroke rehabilitation, suggesting that the functional impact may have varied substantially between patients. Similarly, the increase in VO_2_ peak, while statistically significant, remained modest and should be interpreted primarily as an early functional signal rather than definitive evidence of clinically relevant cardiopulmonary adaptation [34].

Nevertheless, the clinical impact likely varied substantially between individual patients and should be interpreted cautiously given the short follow-up period and observational study design.

### 4.7. Limitations

Several limitations of this study should be acknowledged. First, the relatively small sample size limits the statistical power of subgroup analyses and increases the risk of overfitting in multivariable models. As a result, the regression findings should be interpreted with caution, particularly regarding the identification of independent predictors. The sample size was relatively limited for the number of covariates included in multivariable models, which may have reduced model stability and increased susceptibility to overfitting. Although adjusted R^2^ values were reported to partially account for model complexity, the exploratory nature of these analyses should be emphasized. Second, the absence of a control group precludes definitive conclusions regarding causality. The observed improvements may, in part, reflect natural recovery or the effects of concurrent rehabilitation interventions. Third, the categorization of certain variables, such as stroke location and cardiovascular risk factors, was simplified to ensure adequate statistical power, which may have limited the ability to detect more nuanced associations. Fourth, functional and cardiopulmonary outcomes were assessed over a relatively short follow-up period, which may not fully capture long-term adaptations to treatment. Additionally, because participants were required to ambulate independently and complete CPET testing, the study population represented a relatively higher-functioning subgroup of stroke survivors. Also, the cardiovascular comorbidities within the cohort may have influenced exercise performance independently of spasticity reduction, and residual confounding cannot be completely excluded despite statistical adjustment. Therefore, the findings may not be generalizable to patients with severe disability or marked mobility impairment.

Furthermore, repeated cardiopulmonary exercise testing may have introduced a potential test–retest or familiarization effect, which could have contributed partially to the observed improvements.

In addition, the very high explanatory power observed in some regression models may partially reflect overfitting and regression to the mean, particularly given the relatively limited sample size and the strong dependence of post-treatment outcomes on baseline measurements.

Finally, multiple statistical comparisons were performed without formal adjustment for multiplicity, increasing the possibility of type I error. Consequently, some statistically significant findings should be interpreted cautiously.

### 4.8. Future Perspectives

Future studies with larger sample sizes and controlled designs are needed to confirm these findings and further elucidate the relationship between spasticity management and cardiopulmonary performance. In particular, randomized controlled trials could help determine the extent to which BoNT-A contributes independently to improvements in exercise capacity.

Additionally, incorporating more detailed assessments of autonomic function, energy expenditure, and gait biomechanics may provide deeper insights into the mechanisms underlying the observed improvements.

Finally, longitudinal studies evaluating long-term outcomes and the interaction between BoNT-A therapy and structured rehabilitation programs could help optimize treatment strategies for post-stroke patients.

## 5. Conclusions

Botulinum toxin A treatment for post-stroke spasticity was associated with modest but consistent improvements in cardiopulmonary exercise capacity and functional performance. Significant increases in VO_2_ peak, anaerobic threshold, exercise duration, and maximal workload were accompanied by improvements in walking performance and functional independence. These findings suggest that spasticity reduction may be associated with improvements extending beyond local neuromuscular effects and may contribute to enhanced efficiency of movement and exercise tolerance. Further studies are warranted to confirm these findings and to explore the long-term impact of spasticity management on cardiovascular performance and functional recovery.

## Figures and Tables

**Table 1:** Main characteristics of the population.

Variable	N (%)	Average (SD)/Median (IQR)
Sex		
Male	25 (50%)	
Female	25 (50%)	
Age		74.02 (±10.44) years
BMI		26.76 (±5.14) kg/m^2^
HBP	41 (82%)	
DM	18 (36%)	
CAD	14 (28%)	
Dyslipidemia	29 (58%)	
Smoking status		
Never smoked	24 (48%)	
Former smoker	16 (32%)	
Active smoker	10 (20%)	
Stroke location		
Right-sided	32 (64%)	
Left-sided	16 (32%)	
Other/Bilateral	2 (4%)	
Stroke duration		8.5 (4.0–13.25) months

## Data Availability

The data presented in this study are available on reasonable request from the corresponding author. The data are not publicly available due to privacy and ethical restrictions.
